# Osteopontin accumulates in basal deposits of human eyes with age-related macular degeneration and may serve as a biomarker of aging

**DOI:** 10.1038/s41379-021-00887-7

**Published:** 2021-08-13

**Authors:** Michael Lekwuwa, Mayur Choudhary, Eleonora M. Lad, Goldis Malek

**Affiliations:** 1 Duke Eye Center, Department of Ophthalmology, Duke University School of Medicine, Durham, NC USA; 2 Department of Pathology, Duke University School of Medicine, Durham, NC USA

**Keywords:** Inflammation, Biomarkers

## Abstract

A common clinical phenotype of several neurodegenerative and systemic disorders including Alzheimer’s disease and atherosclerosis is the abnormal accumulation of extracellular material, which interferes with routine cellular functions. Similarly, patients with age-related macular degeneration (AMD), the leading cause of vision loss among the aged population, present with extracellular lipid- and protein-filled basal deposits in the back of the eye. While the exact mechanism of growth and formation of these deposits is poorly understood, much has been learned from investigating their composition, providing critical insights into AMD pathogenesis, prevention, and therapeutics. We identified human osteopontin (OPN), a phosphoprotein expressed in a variety of tissues in the body, as a newly discovered component of basal deposits in AMD patients, with a distinctive punctate staining pattern. OPN expression within these lesions, which are associated with AMD disease progression, were found to co-localize with abnormal calcium deposition. Additionally, OPN puncta colocalized with an AMD risk-associated complement pathway protein, but not with apolipoprotein E or vitronectin, two other well-established basal deposit components. Mechanistically, we found that retinal pigment epithelial cells, cells vulnerable in AMD, will secrete OPN into the extracellular space, under oxidative stress conditions, supporting OPN biosynthesis locally within the outer retina. Finally, we report that OPN levels in plasma of aged (non-AMD) human donors were significantly higher than levels in young (non-AMD) donors, but were not significantly different from donors with the different clinical subtypes of AMD. Collectively, our study defines the expression pattern of OPN in the posterior pole as a function of disease, and its local expression as a potential histopathologic biomarker of AMD.

## Introduction

Age-related macular degeneration (AMD), is an ocular neurodegenerative disease of aging, and the most common cause of irreversible blindness in the elderly in developed countries^1–3^, with consequent detrimental effects on physical and mental health^4,5^. Clinically, the initial stage of AMD development, known as early nonexudative, is characterized by accumulation of lipid- and protein-filled extracellular deposits, basal to the retinal pigment epithelium (RPE)^6–8^, a cuboidal epithelial cell layer that serves as support for everyday functions of the overlying neurosensory retina^9,10^. The basal deposit family includes *drusen*, *basal laminar*, and *basal linear* deposits, all of which are detrimental to RPE function, enable RPE and photoreceptor cell atrophy, and importantly lead to progressive and more severe visual deterioration in the form of advanced nonexudative AMD, also known as geographic atrophy (GA)^11–13^ and the advanced “wet” or neovascular form of AMD^14–16^. Due to the increase in life expectancy, it is predicted that the prevalence of AMD in the aged population will be an even greater health problem in the near future^17^, thus necessitating further investigations into the composition of basal deposits and evolution of the early events in AMD development.

Basal deposit composition along with genetic risk factors identified from epidemiological and genome wide association studies, implicate multiple pathogenic pathways associated with AMD development and progression, underscoring the multifactorial nature of the disease^18–20^. Components of basal deposits identified to date via proteomics and histological methods include factors involved in lipid metabolism and transport, glycoproteins, mineral constituents, and extracellular matrix molecules to name a few^21–24^. Based on these findings, chronic inflammation has emerged as a pathogenic mechanism that may contribute to formation and accumulation of basal deposits, a thought given further credence by the observed localization of several factors associated with immune regulation, including members of the complement pathway, within these lipid- and protein-rich foci^25–28^.

Osteopontin (OPN) is a matricellular protein expressed by a number of cell types throughout the body, including but not limited to macrophages, endothelial cells, epithelial cells, and fibroblasts^29–34^. In the eye, OPN has been reported to be expressed in retinal ganglion, microglial, RPE and choroidal endothelial cells^35–39^. The regulation of osteopontin, which is encoded by the gene secreted phosphoprotein 1 (*SPP1*), is still poorly understood, but studies have indicated that it plays a critical role in several physiologic and pathologic events including mineralization, apoptosis, angiogenesis, cellular remodeling, and inflammation^40–44^. From a disease perspective, OPN has been investigated in neurodegenerative diseases that share common pathogenic pathways with AMD, including Alzheimer’s disease, atherosclerosis, and multiple sclerosis, and found to be differentially regulated in response to either inflammation or injury^45^. Specifically, OPN has been shown to stimulate the expression of pro-inflammatory mediators, triggering relapses in multiple sclerosis^46^, and an increase in inflammatory cells within atherosclerotic plaques^47–49^. Paradoxically, OPN may also mediate anti-inflammatory effects by attenuating oxidative stress through its repression of inducible nitric oxide synthase^50–52^. OPN is also elevated in the cerebral spinal fluid of Alzheimer’s disease patients^53^ suggesting that it may act as a biomarker for the disease. At the molecular level, OPN binds to integrins and CD44 variants on the cell surface to influence cell survival and apoptosis, inflammation, microcalcification, cell attachment and migration, and chemotaxis after an injury^31,46,54^, cellular events also involved to different degrees in AMD development^7,13^.

Given the association between OPN in neurodegenerative diseases that share common pathogenic pathways with AMD as well as OPN’s asserted role in inflammation and macrophage recruitment, herein the expression of OPN was investigated in the context of the pathobiology of AMD. Localization and expression of OPN in eyes from normal and AMD donor tissue were investigated. Mechanistically, the impact of AMD-relevant stressors on extracellular OPN secretion from human RPE cells, cells vulnerable in AMD, was evaluated. Finally, given that age is a major risk factor for AMD, OPN levels in the plasma from young versus old non-AMD donors, were measured along with circulating levels of OPN as a function of clinical stages of AMD. We report that while age effects circulating levels of OPN, local OPN expression correlates with calcified, large drusen, and serves as a histopathologic biomarker of early AMD.

## Methods

### Human ocular tissues

Use of human donor eyes for research was approved by the Duke University Institutional Review Board (Exempt Review), and collected by either BioSight, the North Carolina Organ Donor and Eye Bank Inc., the Alabama Eye Bank, or through Duke Department of Pathology. Eyes used for harvesting ocular cells were processed on ice within 8 h postmortem, as previously described^55–57^, and included fresh isolation of human RPE cells, retina, and choroid. “Non-AMD” donor eyes did not exhibit evidence of retinal/RPE changes upon postmortem evaluation of the posterior pole under a dissecting microscope with back light illumination (Leica MZ6 Stereomicroscope, Leica Microsystems Inc. Buffalo Grove, IL, USA). For histological studies, donor eyes were also collected from patients diagnosed with AMD, the pathologies of which were confirmed on postmortem evaluation of retinal cross-sections stained with hematoxylin and eosin (Table 1 and Supplementary Fig. 1; *n* = 11), with a recorded death to preservation time of 3–44 h. Eyes were preserved by immersion in 4% paraformaldehyde. Approximately 16 h later the anterior segments were removed, posterior poles were embedded in paraffin, and sectioned from the superior cup through the optic nerve to the inferior cup in 10 μm increments^56,58,59^.

**Table 1:** Demographic information of donors for immunohistochemical staining.

Autopsy#	Age	Sex	Race	Postmortem (Hr)	Medical history	Ophthalmic history	Autopsy ophthalmic diagnoses	Majot autopsy systemic diagnoses	Postmortem pathology in retinal cross-sections
AMD-00	87	F	NA	>3 h	NA	NA	Early AMD Eye lift	Cerebrovascular accidnet Shortness of breath Hyperlipidemia Osteoarthritis	Macular and peripheral focal and diffuse basal deposits
0750-15	NA	NA	NA	>6 h	NA	NA	NA	NA	Macular and peripheral focal and diffuse basal deposits
1953-15	NA	NA	NA	NA	NA	NA	NA	NA	Hard macular and diffuse peripheral basal deposits
2618-15	NA	NA	NA	>6 h	NA	NA	NA	NA	Macular and peripheral focal and diffuse basal deposits
AD-03-140	81	F	W	7	Pleural effusion, pulmonary fibrosis, and hypertension Dementia Deep venous thrombosis Breast cancer	None in EMR	Cataract extraction/prosthetic intraocular lens Early AMD	Diffuse alveolar damage Acute bronchopneumonia Atheroclerosis Subendocardial infarcts	Macular and peripheral focal and diffuse basal deposits
AD-04-074	69	M	B	27.3	Metastatic prostate cancer Multiorgan failure after liver biopsy causing massive hemorrhage	None in EMR	Early AMD Chronic nongranulomatous choroiditis Retinal hemmorrhages	Status post exploratory Centrilobular necrosis of the liver Metastatic prostate adenocarcinoma Acute and subacute myocaridal infarcts	Macular and peripheral focal and diffuse basal deposits RPE clumping and atrophy
AD-04-100	84	F	W	23	Cerebrovascular disease Supraventricular arrhythmia Hypertension and nephropathy, renal insufficiency Coronary artey disease Esophageal dysmotility CHF and hyperlipidemia Diabetes	None in EMR	Atrophic “dry” AMD Corneal edema Cataract extraction/prosthetic intraocular lens	Hematoma and hemorrhage Cerebrovascular and coronary atherosclerosis Emphysema Pancreatitis Diverticula of transverse, discending, and sigmoid colon Diabetic nephropathy of bilateral kidneys Diffuse glomerular sclerosis	Peripapillary deposits Macular and peripheral basal deposits Geographic atrophy Neovascular wet AMD
AD-04-133	80	M	B	6	Hypertension Chronic renal insufficiency Hypothyroidism and hyperlipidemia AV block and pacemaker Amyloidosis	None in EMR	Early AMD Chronic nongranulomatous choroiditis Retinal hemmorrhages	Systemic amyloidosis Left ventricular hypertrophy Pleural effusions Coronary atherosclerosis and mild cerebrovascular atherosclerosis	Macular peripheral basal deposits RPE pigmentory changes Geographic atrophy
AD-05-25	NA	NA	NA	>6 h	NA	NA	NA	NA	Macular and peripheral focal and diffuse basal deposits
AD-06-070	100	F	W	44	Alzheimer disease Cataract surgery Extremely hard of hearing	None in EMR	Atrophic (“dry”) AMD Confluent drusen Fuchs’ corneal dystrophy Bullous keratopathy Prosthetic intraocular lens Pseudoadenomatous hyperplasia of the ciliary epithelium Chronic conjunctivitis Calcified senile scleral plaque	Alzheimer’s disease Midoccipital infarct Moderate cerebrovascular atherosclerosis Acute bronchiolitis Pedunculated adenomatous polyp of the trasnverse colon Extensive scarring of the pancreas typical of remote pancreatitis	Macular and peripheral focal and diffuse basal deposits
AD-06-227	88	M	W	13	Atrial fibrillation and ischemic cardiomyopathy Hypertension Diabetes	None in EMR but patient on Xalatan for glaucoma	Atrophic (“dry”) AMD Atrophy of the right and left optic nerves Confluent drusen with photoreceptor degeneration Cataract extraction/prosthetic intraocular lens Calcified senile scleral plaques Choroidal infarcts	Status post left above-the-knee amputation Atherosclerosis of the coronary ateries Occlusion of multiple coronary arteries Fibrosis of the interventricular septum Acute centrilobular necrosis of the liver Parkinson’s disease Alzheimer’s disease Sagittal sinus thrombosis Moderate cerebrovascular atherosclerosis	Geographic atrophy, Macular and peripheral deposits Retinal atrophy Neovascularization

*NA* Not available, *AMD* age-related macular degeneration, *EMR* electronic medical records, *RPE* retinal pigment epithelium.

### Cell culture lines

In vitro assays included the use of primary RPE (hRPE) cells harvested from human non-AMD donor eyes (*n* = 3; 48-year-old male, passages 2–4; 84-year-old male, passage 2–4; and 93-year-old female, passages 4–7), the human derived ARPE19 cell line (passages 26–28), and the macaque derived RF/6A choroidal endothelial cell line (passage 32–35), cultured as previously described^57,59,60^. In vitro experiments were performed on RPE cell cultures grown to a post-confluent state with demonstrated zonula occludens-1 immunoreactivity in parallel wells^55–58^.

### RNA isolation and qPCR

Total RNA isolated from cultured cells, freshly isolated human RPE cells, retina, and choroid, along with RNA quality assessment, cDNA reverse transcription, and PCR, were completed as previously described^55–58^. The death to preservation time for the human donor tissue was <8 h. PCR was performed using the Bio-Rad CFX96 Real-time PCR Detection System (Bio-Rad, Hercules, CA). The amplification products of *OPN and 36B4* primers obtained after Real time PCR were run on a 1% agarose gel and visualized with ethidium bromide. Primer sequences used were selected from Primer Bank, http://pga.mgh.harvard.edu/primerbank. The primer sequences are as follows: OPN: Forward—5′-CTCCATTGACTCGAACGACTC-3′, Reverse—5′-CAGGTCTGCGAAACTTCTTAGAT-3′, and 36B4: Forward—5′-GGACATGTTGCTGGCCAATAA-3′, Reverse—5′-GGGCCCGAGACCAGTGTT-3′.

### Protein isolation and western blot protocol

Protein was isolated from cell culture and human tissue (retina, RPE, and choroid) and total protein was determined using the Pierce BCA Protein Assay Kit (Thermo Fisher Scientific, Waltham, MA), as previously described^55^. The death to preservation time for the human donor tissue was <8 h. Western blotting was performed as previously described with the housekeeping protein, beta-actin (Table 2), serving as a loading control^58^.

**Table 2:** Antibodies/stains used for western blot, immunofluorescence, and histology.

Antibody/stain	Source	Dilution	Catalog#
Alpha-Smooth muscle actin (αSMA)	Sigma-Aldrich	1:5000	A5228
Apolipoprotein E (APOE)	Millipore	1:500	AB947
Beta-Actin	SCBT	1:5000	sc-47778
CD31	Dako	1:50	M0823
Complement factor H (CFH)	Quidel	1:200	A312
Hoechst 33258 pentahydrate	Invitrogen	2 μg/ml	H3569
Iba1	Wako	1:200	019-19741
Osteopontin (OPN)	Sigma-Aldrich	1:200	O7264
Vitronectin (VTN)	Santa-Cruz	1:500	SC-74484
Von-Kossa method for calcium kit	Polysciences		24633-1

### Immunohistochemistry

Protein localization in paraffin sections probed with antibodies (Table 2), was performed as previously described^55,59,60^. Nonspecific immunostaining in sections was blocked with normal serum (Jackson Immunoresearch, West Grove, PA, USA) appropriate to the secondary antibody species. Secondary antibodies were conjugated to AlexaFluor 568 or 488 (Invitrogen, Carlsbad, CA). Control slides containing sequential sections were probed with nonimmune serum and buffer without primary antibody. Paraffin sections were subjected to deparaffinization and antigen retrieval to restore the immunoreactivity of epitopes, prior to initiation of the immunohistochemistry staining procedure^59^. Images were visualized and collected using a Nikon C2si confocal microscope Nikon Instruments Inc., Melville, NY, and processed using the Nikon Elements software (AR 4.50.00 64-bit) to display maximum intensity projection.

### Peptide competition immunofluorescence assay

To further demonstrate specificity of the OPN antibody, immunofluorescence staining was performed in the presence of recombinant human peptide (rhOPN). Briefly, the OPN antibody was incubated for 24 h with rhOPN (#1433-OP; R&D systems, Minneapolis, MN) at two different antibody-to-peptide ratios (1:5 and 1:10), followed by immunostaining as described. The sections were also subjected to autofluorescence quenching by Vector^®^ TrueVIEW^®^ Autofluorescence Quenching Kit (Vector Laboratories, Inc. Burlingame, CA) to reduce RPE autofluorescence.

### OPN immunoreactivity quantification

Immunohistological images of OPN stained retinal cross-sections were processed using Adobe photoshop CC to measure the diameter (largest dimension) of the individual OPN puncta in each druse. The diameter of a total of 79 OPN positive puncta (*n* = 4 slides from *n* = 3 donors) was measured and reported as μm. Slides probed with the OPN antibody were also scanned and the percentage of total number of drusen containing OPN positive puncta was also calculated (*n* = 1 slide from *n* = 7 donors).

### Von-Kossa histochemistry stain

To stain for evidence of calcification in paraffin sections, the “Von-Kossa method for calcium” kit (Polysciences Inc: 3% Silver nitrate, 5% Sodium thiosulfate, and Nuclear fast red), an indirect histochemical stain, was used according to manufacturer’s protocol.

### Cell culture

In a T25 flask, 2 × 10^5^ human primary RPE (hRPE) cells/well were plated in DMEM/F12 medium (Thermo Fisher Scientific) supplemented with 10% fetal bovine serum (FBS; Sigma, St. Louis, MO). Post confluence, cells were serum-starved (1% FBS-DMEM/F12) for 24 h and treated with the following AMD-relevant stressors: 5% Cigarette-Smoke Extract (CSE)^55,61^, 50 μM hydroquinone (HQ; Sigma); 50 μg/mL recombinant OPN (R&D Systems), 20 μM Arachidonic acid (AA; MP Biomedicals, USA), 1 μg/mL Docosahexaenoic Acid (DHA; Sigma-Aldrich,), 1 mM vascular endothelial growth factor (VEGF; R&D Systems), 50 μg/mL oxidized low-density lipoproteins (oxLDL; Invitrogen, Eugene Oregon), 50 μg/mL LDL (Invitrogen), and 100 µM sodium iodate (NaIO3; Sigma-Aldrich) for 48 h. The media was collected and secreted proteins from human RPE cells were concentrated using the Pierce Protein Concentrator Kit (Thermo Scientific), followed by assessment of protein levels using the Pierce BCA Protein Assay kit, prior to measuring OPN levels using the Quantikine Human OPN ELISA kit (R&D Systems) according to the manufacturer’s protocol.

### Cell viability

hRPE cultures were visualized using a Zeiss Observer D1 microscope (Carl Zeiss Microscopy LLC, White Plains, NY) after 48 h treatment with AMD-relevant stressors as described above, then harvested to assess cell viability using CellTiter-Blue^®^ (Promega, Madison, WI), according to the manufacturer’s protocol. The results were reported as relative fluorescence (excitation: 560 nm, emission: 590 nm).

### OPN human donor plasma quantification

Human donor (Table 3) blood samples were collected from young (20–28-year-old) and old (62–86-year-old) non-AMD individuals and AMD patients diagnosed with early AMD (drusen diameter 64–124 μm); intermediate AMD (drusen diameter >125 μm and no evidence of GA), or neovascular AMD under a Duke approved Institutional Review Board protocol (# Pro00063921) in glass tubes containing heparin or EDTA as an anticoagulant. Plasma samples were centrifuged (1000 × *g*) for 15 min at 4 °C, immediately aliquoted, and transferred into new Eppendorf tubes for storage at −80 °C. OPN levels were determined and quantified using the Quantikine Human OPN ELISA kit according to the manufacturer’s protocol (R&D Systems). Optical coherence tomography (OCT) and fundus images were recorded in the clinic as part of the patient’s routine clinical exam.

**Table 3:** Demographic information of plasma donors.

	Study group	Age	Gender	Race	Ethnicity
1	Non-AMD (Young)	20	F	African American	Not Hispanic or Latino
2	Non-AMD (Young)	24	F	White	Not Hispanic or Latino
3	Non-AMD (Young)	25	M	White	Not Hispanic or Latino
4	Non-AMD (Young)	28	M	White	Unknown
5	Non-AMD (Young)	28	F	Asian	Not Hispanic or Latino
1	Non-AMD (Aged)	62	M	White	Not Hispanic or Latino
2	Non-AMD (Aged)	63	M	White	Not Hispanic or Latino
3	Non-AMD (Aged)	64	F	White	Not Hispanic or Latino
4	Non-AMD (Aged)	66	F	White	Not Hispanic or Latino
5	Non-AMD (Aged)	75	Unknown	Unknown	Unknown
6	Non-AMD (Aged)	78	F	White	Not Hispanic or Latino
7	Non-AMD (Aged)	79	Unknown	Unknown	Not Hispanic or Latino
8	Non-AMD (Aged)	81	F	White	Not Hispanic or Latino
9	Non-AMD (Aged)	81	Unknown	Unknown	Unknown
10	Non-AMD (Aged)	85	Unknown	Unknown	Unknown
11	Non-AMD (Aged)	86	Unknown	Unknown	Unknown
12	Non-AMD (Aged)	Unknown	M	White	Not Hispanic or Latino
1	Early-intermediate AMD	59	F	White	Not Hispanic or Latino
2	Early-intermediate AMD	59	M	White	Not Hispanic or Latino
3	Early-intermediate AMD	69	F	White	Not Hispanic or Latino
4	Early-intermediate AMD	76	F	White	Not Hispanic or Latino
5	Early-intermediate AMD	78	F	White	Not Hispanic or Latino
6	Early-intermediate AMD	84	F	White	Not Hispanic or Latino
1	Late AMD	60	Unknown	Unknown	Unknown
2	Late AMD	66	M	White	Not Hispanic or Latino
3	Late AMD	66	F	White	Not Hispanic or Latino
4	Late AMD	66	M	White	Not Hispanic or Latino
5	Late AMD	66	M	White	Not Hispanic or Latino
6	Late AMD	68	F	White	Not Hispanic or Latino
7	Late AMD	69	Unknown	Unknown	Unknown
8	Late AMD	69	F	White	Not Hispanic or Latino
9	Late AMD	70	F	White	Not Hispanic or Latino
10	Late AMD	73	M	White	Not Hispanic or Latino
11	Late AMD	73	F	White	Not Hispanic or Latino
12	Late AMD	75	F	White	Not Hispanic or Latino
13	Late AMD	77	M	White	Not Hispanic or Latino
14	Late AMD	78	M	White	Not Hispanic or Latino
1	CNV	65	F	White	Not Hispanic or Latino
2	CNV	68	F	White	Not Hispanic or Latino
3	CNV	69	F	White	Not Hispanic or Latino
4	CNV	77	F	White	Not Hispanic or Latino
5	CNV	77	F	White	Not Hispanic or Latino
6	CNV	78	F	White	Not Hispanic or Latino
7	CNV	81	F	White	Not Hispanic or Latino

### Statistical methods and rigor

Statistical methods for data analysis included two-tailed Student’s *t* test and two-way ANOVA, followed by Sidak’s multiple comparison test using GraphPad Prism (Version 9.0.0 for Windows, Graphpad Software LLC., San Diego, CA). Values were considered statistically significant at *p* < 0.05. For the in vitro experiments, the technical samples were run in triplicate and biological experiments were performed a minimum of three times.

## Results

### OPN is expressed in the posterior human retina

**Fig. 1:**
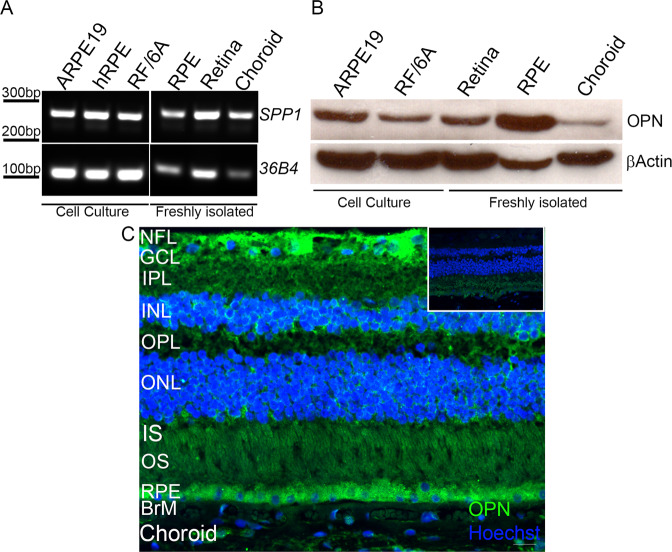
OPN is expressed in cells of the posterior retina and human donor eyes. **A** Real-time PCR of the *SPP1* gene (OPN) in ARPE19, primary RPE cells (hRPE, 93-year-old, female donor), RF/6A cells, and freshly isolated RPE, retina, and choroid tissues from a 74-year-old, female donor. **B** Western blot showing OPN protein expression in ARPE19, RF/6A cells, and freshly isolated retina, RPE, and choroid. **C** Immunofluorescence staining of retinal sections from an aged non-AMD donor shows OPN expression throughout the retinal layers, RPE, and choroid (Scale bar = 20 µm). No primary control is shown in the inset. (BrM Bruch’s membrane, GCL ganglion cell layer, INL inner nuclear layer, IPL inner plexiform layer, IS photoreceptor inner segments, NFL nerve fiber layer, ONL outer nuclear layer, OPL outer plexiform layer, OS photoreceptor outer segments, RPE retinal pigment epithelial cells).

To identify ocular cells and tissue compartments that express OPN, gene expression was examined in cells isolated from the posterior retina, including cultured hRPE cells, the human derived ARPE19 cell line, and the macaque derived RF/6A choroidal endothelial cell line, in addition to freshly isolated RPE, retina, and choroid from human donor eyes. We observed expression of OPN mRNA (*SPP1*) and protein in the in vitro cell model systems as well as in freshly isolated human tissue (Fig. 1A, B), confirming that retina, RPE, and choroid tissue in the posterior pole express OPN and these tissues may potentially act as a local source of OPN. Additionally, we observed protein immunolocalization of OPN in retinal cross-sections from non-AMD donor eyes, throughout the retinal ganglion cell layer, nerve fiber layer, inner plexiform layer of the retina, the RPE, and cells within the choroid (Fig. 1C). Specificity of the anti-OPN antibody was confirmed via western blot evaluation of OPN recombinant protein and immunohistochemistry competition assays (Supplementary Figs. 2, 3).

### OPN expression in early AMD

**Fig. 2:**
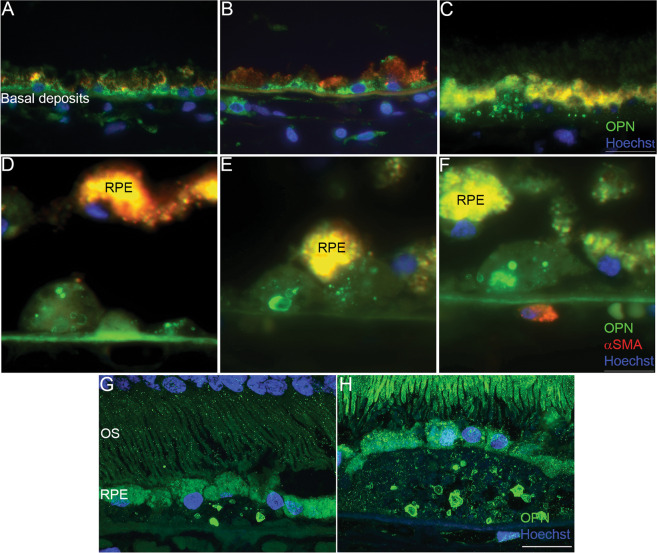
OPN immunoreactivity within basal deposits. **A**, **B**, **C** Immunofluorescence staining of human AMD donor eyes showing OPN puncta within diffuse basal deposits and RPE cells. **D**, **E**, **F**, **G**, **H** High magnification images of AMD donor eyes showing punctate OPN staining within small- and medium-sized drusen. Scale bar (**C**, **H**) = 20 μm, Scale bar (**F**) = 40 μm (OS photoreceptor outer segments, RPE retinal pigment epithelial cells). OPN green in (**A**–**H**); α-smooth muscle actin: red in (**A**–**F**).

**Fig. 3:**
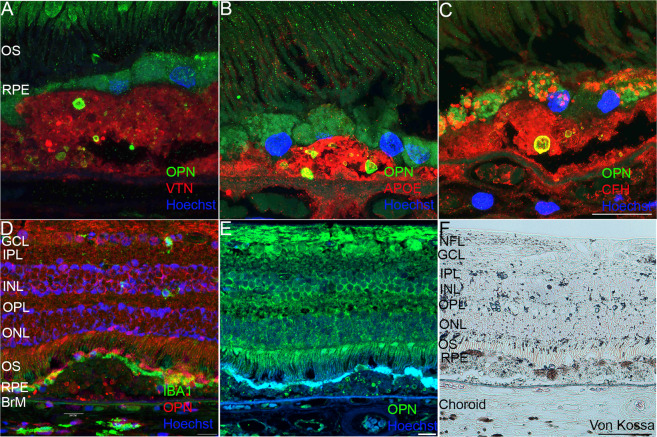
Colocalization of OPN with known drusen components. Immunofluorescence staining of AMD human donor eyes showing the OPN (green) puncta pattern in drusen relative to (**A**) Vitronectin (VTN: red), (**B**) Apolipoprotein E (APOE: red), and (**C**) Complement Factor H (CFH: red). Scale bar in (**C**) = 20 µm. (**D**) OPN immunoreactivity (red) versus the distribution of the microglial marker IBA1 (green) throughout the full neurosensory retina, RPE, and choroid. **E** OPN immunoreactivity (green) (Scale bar = 20 μm) and (**F**) Von-Kossa staining of serial cross-sections ~25 μm apart from an AMD human donor eye, showing calcification (black) and counterstained nuclei (pink). (Scale bar = 20 μm). BrM Bruch’s membrane, GCL ganglion cell layer, INL inner nuclear layer, IPL inner plexiform layer, NFL nerve fiber layer, ONL outer nuclear layer, OPL outer plexiform layer, OS photoreceptor outer segments, RPE retinal pigment epithelium.

Retinal cross-sections from donor eyes diagnosed with early-dry AMD were probed with the antibody to OPN and found to show OPN positive immunoreactivity throughout basal deposits, and drusen of various sizes. OPN positive staining was observed in spherules and were often punctate in nature within thin and thick diffuse basal deposits (Fig. 2A, B, C). Small hard drusen also contained punctate OPN immunoreactivity, presenting as large spherules with more well defined shapes (Fig. 2D, E, F). Noteworthy, is that a lower density of OPN spherules was observed in the hard druse compared to the thin and thick diffuse deposits (Fig. 2). OPN staining in the RPE overlying basal deposits varied with punctate staining present primarily in RPE overlying drusen (Fig. 2C, E, F, H) and less so above basal laminar deposits (Fig. 2A, B). Examination of medium–large sized (Fig. 2G, H), revealed a larger concentration of OPN positive spherules within large drusen. Overall, greater than 68% of drusen contained OPN positive spherules to some degree. These OPN positive spherules appeared to be well demarcated with a cross-sectional size ranging from 0.5 to 15 μm in diameter. We also probed retinal sections from AMD donors with antibodies against known protein components of drusen and found that drusen showed positive staining for vitronectin (VTN) throughout the deposit, which did not co-localize with OPN (Fig. 3A). Similarly, apolipoprotein E (APOE) was observed to be diffuse throughout the druse and within the choriocapillary pegs (Fig. 3B), while complement factor H (CFH) localized within the RPE, druse, and the choroid and overlapped with OPN positive spherules (Fig. 3C). Finally, given the reported relationship between OPN and macrophage recruitment and retention, we examined the staining pattern of Iba-1, a microglia/macrophage calcium-binding protein^58,62^ relative to that of OPN. Iba-1 immunoreactivity was evident within the neural retina, the choriocapillaris, and adjacent to the RPE overlying drusen containing OPN positive puncta (Fig. 3D).

### OPN puncta localize within calcified drusen in donor AMD eyes

OPN is aspartic acid-rich, which makes it a highly acidic protein. This feature, combined with the presence of putative calcium (Ca^2+^) binding motifs, allows it to bind large amounts of Ca^2+^ and to interact with hydroxyapatite crystals with high affinity^63^. Moreover, calcification in drusen has been identified in AMD-affected eyes by multiple modalities and has been associated with disease progression^64–66^. We examined the relative staining pattern of OPN within calcified drusen in AMD donor eye tissue by staining adjacent paraffin sections (~25 μm apart) probed for OPN, with the Von-Kossa histochemical stain. We found that whereas non-AMD eyes did not display any calcified particles in the posterior retina (data not shown), extensive calcifications were seen throughout large drusen in a punctate manner similar to the OPN spherules, suggesting a possible role of OPN in accumulation of Ca^2+^ ions in the drusen (Fig. 3E).

### AMD-relevant stressors can stimulate OPN secretion from RPE cells

**Fig. 4:**
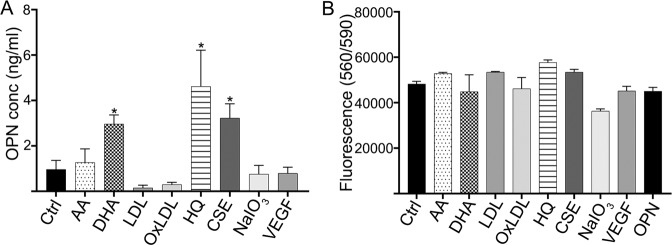
Extracellular OPN levels secreted from human primary RPE cells in response to AMD-risk factors. (**A**) OPN levels were measured by ELISA in media collected from human primary RPE cells, from a 93-year-old donor, treated with AMD-risk factors. (*p < 0.05, *n* = 3, One-way ANOVA, multiple comparisons). (**B**) Cell viability of hRPE cells in response to treatment. (AA arachidonic acid, DHA docosahexaenoic acid, LDL low-density lipoprotein, OxLDL oxidized LDL, CSE cigarette-smoke extract, HQ hydroquinone, NaIO3 sodium iodate, VEGF vascular endothelial growth factor.

To determine mechanisms that may underly extracellular deposition of OPN from RPE cells, we tested the ability of hRPE cells to secrete OPN following exposure to AMD-stressors. Of the three hRPE cells lines tested, RPE cells derived from the 93-year-old donor were found to be capable of secreting OPN protein, as measured by ELISA, in response to selective injury sublethal treatments including the oxidants, cigarette-smoke extract (CSE; 2.7 ng/ml) and hydroquinone (HQ: 4.3 ng/ml), and the omega fatty acid, docosahexaenoic acid (DHA; 2.5 ng/ml; Fig. 4A). Sublethal levels of the treatments were confirmed by morphology and cell viability assessments (Supplementary Figs. 4,  4B). The baseline levels of OPN secretion from the other two cells lines derived from a 48 year old and 84 year old were close to zero. Sublethal injury of the young cell line with AMD-risk factors did not induce OPN secretion, while treatment with cigarette-smoke extract and hydroquinone was able to stimulate a low level of OPN secretion (0.54 and 0.6 ng/ml, respectively) from the RPE cells collected from the 84 year old. These findings while highlighting subjective differences in response to injury, support that aged RPE cells may be a local ocular source of OPN and that select AMD-risk factors are capable of triggering OPN secretion from RPE cells leading it its accumulation in basal deposits.

### Plasma OPN levels increase in human donors as a function of age

**Fig. 5:**
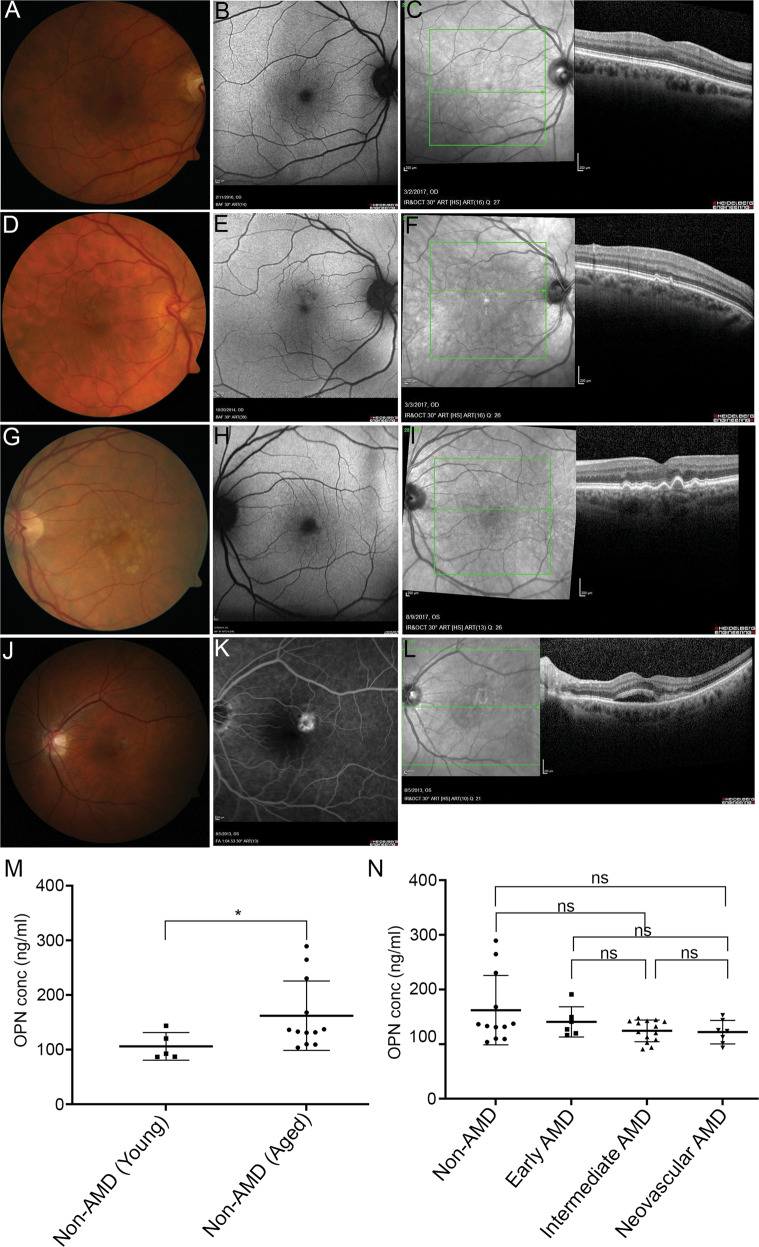
Systemic OPN levels in AMD patients. Representative donor fundus photos (**A**, **D**, **G**, **J**), fundus autofluorescence (**B**, **E**, **H**, **K**), and OCT images (**C**, **F**, **I**, **L**) for different AMD disease stages; (**A**, **B**, **C**) Non-AMD (Aged), (**D**, **E**, **F**) Early AMD, (**G**, **H**, **I**) Intermediate AMD, and (**J**, **K**, **L**) Neovascular AMD (Scale bar = 200 μm). Plasma OPN levels measured by ELISA in plasma of (**M**) Non-AMD (Aged) versus Non-AMD (Young) and (**N**) AMD patients classified as early, intermediate, and neovascular AMD with choroidal neovascularization. (*p < 0.05, One-way ANOVA, Sidak’s multiple comparisons).

To determine if systemic OPN levels vary with age and AMD disease status, we measured OPN concentrations in plasma collected from human donors. We found that non-AMD donors exhibited an age-related increase in systemic OPN levels as a function of age. Noteworthy, however is that only three individuals in the aged group showed elevated OPN levels (>235 ng/ml), resulting in a statistically significant difference (Fig. 5). We did not observe any significant differences in OPN levels as a function of severity of disease in AMD patients with early, intermediate, and neovascular AMD. Intermediate AMD and CNV showed a trend towards a drop in OPN levels, though this was most likely due to a few aged donors with elevated circulating OPN levels.

## Discussion

Much of our understanding of the pathology of ocular diseases such as AMD, in recent years, has relied on OCT imaging of patients^67,68^. However, evaluation of postmortem ocular tissue remains the gold standard for ascertaining biochemical composition and protein localization in tissue and subcellular regions. Through histochemistry and immunohistochemistry many of the components of basal deposits, characteristic lesions of early AMD, which are also risk factors for progression of AMD, have been identified to date, including but not limited to, esterified and unesterified cholesterol, cholesterol carriers including apolipoproteins, clusterin, vitronectin, components of the complement pathway, and proteoglycans^11,22,66,69,70^. Knowledge of these molecules, in turn, have led to confirmatory support for genetic risk factors identified and/or have revealed pathogenic pathways that may be involved in the initiation and progression of AMD, including inflammation, dysregulation of lipid transport and processing, and alternations in extracellular matrix homeostasis, thus underscoring the value of identifying tissue biomarkers of early AMD.

Herein we identified and localized the phosphoprotein, OPN, within the inner and outer human retina and discovered it to be a component of basal deposits. We also observed that though this inflammatory mediator’s punctate pattern parallels that of calcification and complement factor H in drusen and basal laminar deposits of a variety of sizes, it does not co-localize with other known drusen components including apolipoprotein E and vitronectin, which are diffusely spread throughout the deposits^22^. Furthermore, we examined potential local mechanisms contributing to extracellular deposition of OPN and found AMD-associated risk factors including oxidant injury and lipid challenge, capable of upregulating OPN secretion from RPE cells derived from aged donors. Finally, while systemically OPN levels varied with advanced age, no differences were detected based on disease clinical sub-type.

Inflammation and the recruitment of immune cells creating a pro-inflammatory microenvironment in the subretinal space, have consistently been implicated in the development and progression of AMD^13,71–73^. Our discovery of OPN as potential tissue biomarker of AMD, since it accumulates in extracellular lesions characteristic of early AMD, further supports the reported role that inflammation plays in the pathogenesis of the disease. However, the role of OPN in the posterior segment of the eye may not be limited to inflammation as it has also been implicated in fibrosis and chemotaxis^74,75^, raising the possibility that extracellular OPN accumulation may reflect RPE differentiation (epithelial-to-mesenchymal transition)^32^, as OPN was originally identified as a secreted protein from transformed epithelial cells^76^. Importantly, the presence of OPN in the choroid may hint to its involvement in not only macrophage recruitment, but also endothelial-to-mesenchymal transition and/or endothelial migration^32,77^. Though exposure to vascular endothelial growth factor (VEGF) in RPE cells in culture in our study did not result in elevated OPN, there are studies that indicate OPN can promote angiogenesis and endothelial migration though PI3K/AKT pathways with VEGF acting as a positive feedback signal^78^.

Mechanistically, another physiological responsibility of OPN is control of biomineralization, through its ability to bind apatite crystals^31^. OPN knockout mice reportedly present with hypermineralized and fragile bones^79^. Moreover, OPN has been shown to accumulate and co-localize in regions with pathological calcification in atherosclerotic lesions, aortic stenosis, kidney stones, and the vasculature^34,63,80,81^. Of relevance to AMD, are studies demonstrating the accumulation of hydroxyapatite spherules within drusen^82^, in a similar pattern to that of OPN as observed in our study. We also made the observation that OPN puncta were found in calcified drusen, an indicator of AMD progression^65,83–85^. OPN contains an integrin binding arginine-glycine-aspartate (RGD) motif and aspartic-acid rich calcium-binding domains, which may facilitate its localization and accumulation in drusen, as drusen itself is a conglomerate of lipids and proteins. In fact, vitronectin, a well-established marker of drusen^86^, contains RGD motifs, as well as calcium rich hydroxyapatite^65,82^, thus providing OPN a framework and the physical forces required for its build up and accumulation. The complex physical interactions of OPN and drusen components need to be investigated thoroughly to further tease out the molecular crosstalk in deposits.

There is evidence for both local and systemic sources of OPN based on studies of a variety of tissues and cells including macrophages, activated microglia, astrocytes and epithelial cells^33,87–92^, as well as neurodegenerative diseases such as multiple sclerosis, Alzheimer’s disease, Parkinson’s disease, and ischemic stroke^53,91,93–97^. In the human donor eyes, OPN expression was detected in freshly isolated tissue from RPE, choroid, and retina, suggesting multiple tissues in the posterior retina express, and have the potential to secrete OPN. Challenging primary human RPE cells from aged donors in culture with AMD-relevant stressors, supported select oxidative insults including cigarette-smoke extract and hydroquinone, as well as increased lipid burden as potential inducers of OPN secretion, locally. The triggers for OPN secretion by other retinal cells remain to be identified. Though the role of OPN is tissue specific, these results are congruent with mouse studies in which OPN levels have been shown to increase during the onset of drug-induced liver injury^98^.

The levels of OPN in systemic circulation were also measured and found to increase with aging in the plasma. Noteworthy, was the presence of two populations in the aged group, one in which OPN levels were similar to the young donors, and the other in which OPN levels were elevated. The small cohort size of donors in our study is a limitation and it is plausible that with a larger cohort, this potential difference may be evaluated further and allow for correlation studies taking into consideration the inflammatory status of the two groups and testing the hypothesis that in a subpopulation of individuals, OPN may be transported via the choroidal circulation to the sub-RPE space. Interestingly, when OPN levels were measured as a function of AMD progression, statistically significant differences were not detected and in fact the levels were similar to that of young donors. Overall, though it is possible that an additional source of OPN may come from the circulation, our findings so far are supportive of local OPN production.

Our discovery of large numbers of OPN immunopositive puncta in deposits characteristic of AMD, is a novel finding, however the function of OPN in the sub-RPE space remains to be fully elucidated. On the one hand it has been suggested that OPN has neuroprotective and repair-promoting effects in neurodegenerative diseases such as Alzheimer’s disease and considering the parallels between AMD and neurodegenerative diseases, it is possible that OPN might be induced and secreted from RPE cells in a last-ditch effort to preserve its function^99–101^. On the other hand, OPN secretion might serve as a cytokine, which is involved in the recruitment of activated microglia/macrophages in the immune-privileged subretinal space in response to oxidative injury to the RPE cells. Understanding the distribution of the alternative splicing products of the human OPN, which generates three different isoforms (OPNa, OPNb, and OPNc) will also be important, as these isoforms contribute to the variable expressions and functional properties of OPN in different pathophysiological conditions. Additionally, though in humans, most studies on OPN have primarily focused on the secreted protein, OPN is also known to undergo posttranslational modifications and the role of intracellular OPN in RPE cells remains to be considered. Finally, future studies focused on the role of OPN in the choroid and retina, tissues also vulnerable in AMD, along with animal models in which local OPN levels have been altered will be necessary in order to shed further light on the role of OPN in drusen biogenesis, RPE cell function, and AMD progression.

## Supplementary information

All Supplementary material in PDF

## Data Availability

The datasets used and/or analyzed during the current study are available from the corresponding author on reasonable request.
