# Supplementary material for: Osteopontin accumulates in basal deposits of human eyes with age-related macular degeneration and may serve as a biomarker of aging
Source: Mod Pathol. 2021 Aug 13;35(2):165–76. doi: 10.1038/s41379-021-00887-7 (PMC8786662; doi:10.1038/s41379-021-00887-7)
Supplement: Supplementary file 1 — All Supplementary material in PDF [file 41379_2021_887_MOESM1_ESM.pdf]

Supplementary Information

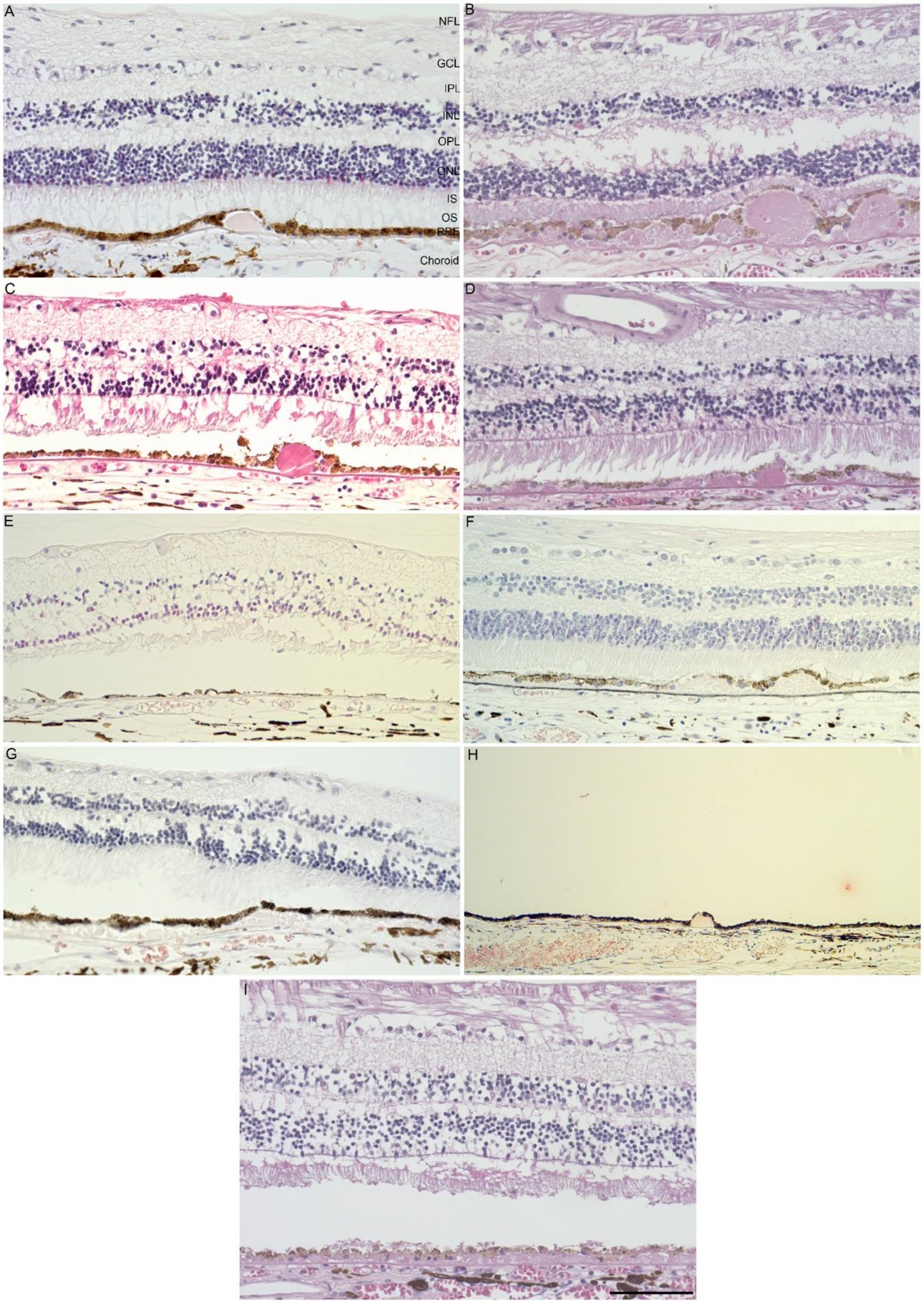

Supplementary Figure 1 Human donor tissue samples used for immunohistology. Representative images of hematoxylin and Eosin (H&E) staining of different human donor tissue samples used in this study (Scale bar = 20 μm). (A) 0750-15, (B) AD03-140, (C) AD04-133, (D) AD06-070, (E) AD06-227, (F) AMD-00, (G) 2618-15, (H) 1953-15 (retina detached), and (I) AD04-074 (GCL: Ganglion Cell Layer, INL: Inner Nuclear Layer, IPL: Inner Plexiform Layer, IS: Photoreceptor Inner segments, NFL: Nerve Fiber Layer, ONL: Outer Nuclear Layer, OPL: Outer plexiform layer, OS: Photoreceptor Outer Segments, RPE: Retinal Pigment Epithelial cells).
.

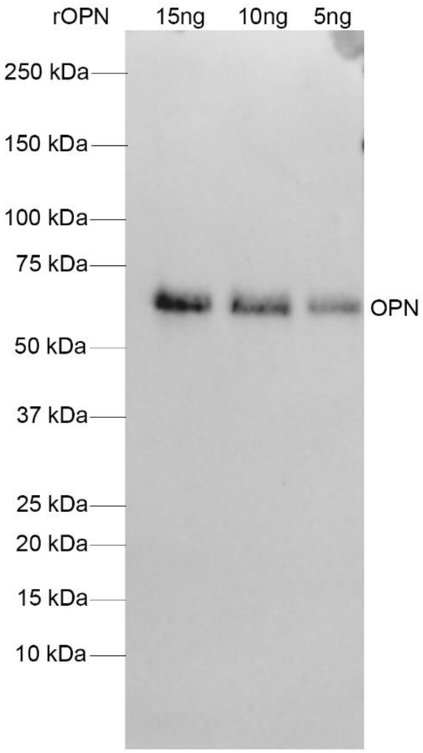

Supplementary Figure 2 Western blot demonstrating specificity of OPN antibody using serial concentrations of recombinant human OPN.

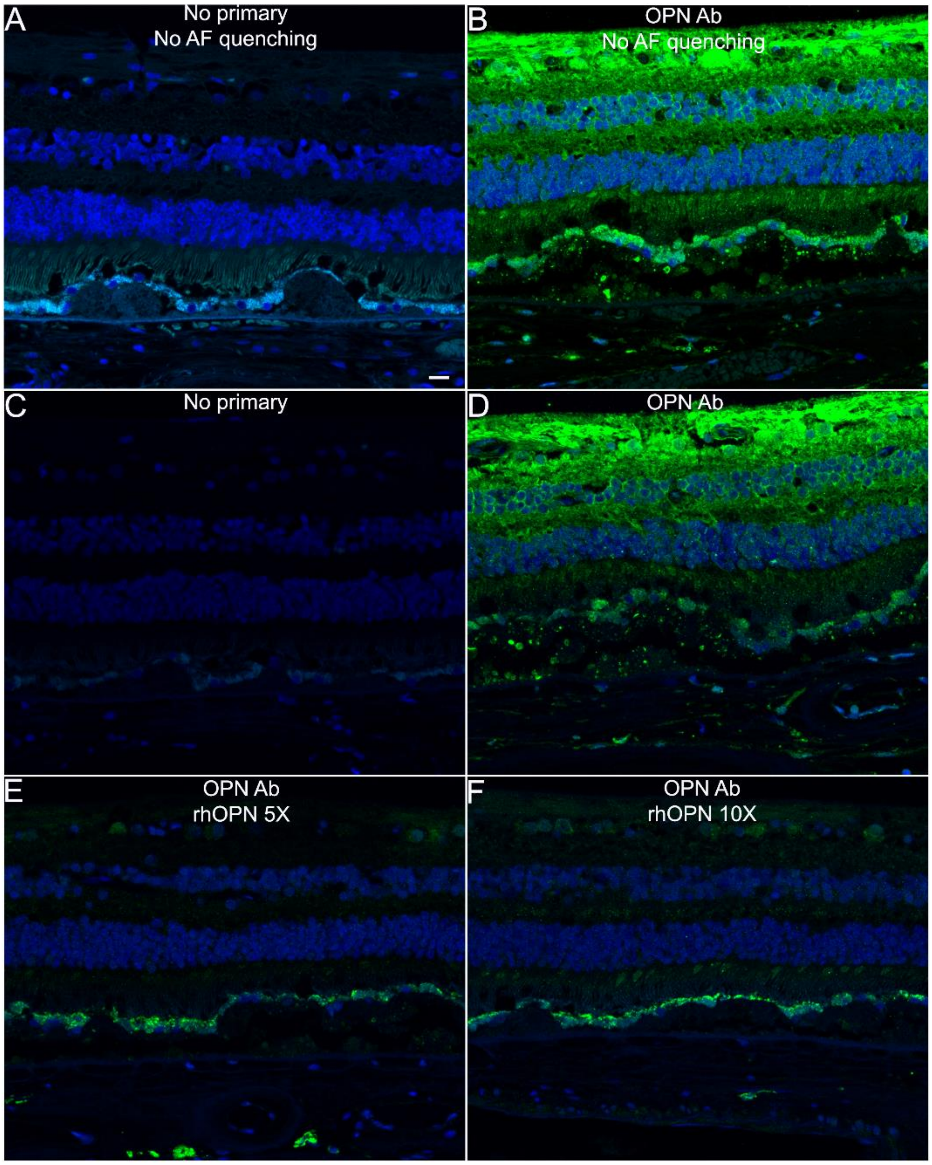

Supplementary Figure 3 rhOPN peptide blocks staining by OPN antibody: Representative images from peptide competition immunofluorescence staining using OPN antibody and rhOPN. (A) no primary control, no autofluorescence (AF) quenching (B) OPN staining, no AF quenching, (C) no primary control, (D) OPN antibody only, (E) OPN antibody and rhOPN (1:5), and (F) OPN antibody and rhOPN (1:10) (Scale bar = 20 µm). AF quenching was performed using Vector® TrueVIEW® Autofluorescence Quenching Kit in panels C, D, E and F.

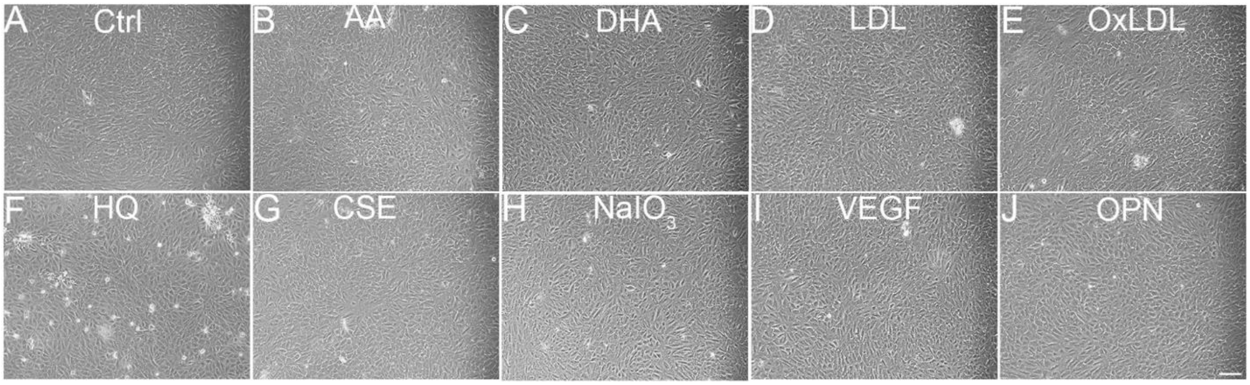

Supplementary Figure 4 *In vitro* RPE injury treatments. Representative images showing RPE cell morphology after treatment with AMD-stressors and recombinant OPN. (A) Vehicle Control, (B) AA: Arachidonic Acid, (C) DHA: Docosahexaenoic acid, (D) LDL: Low-Density Lipoprotein, (E) OxLDL: Oxidized LDL, (F) HQ: Hydroquinone, (G) CSE: Cigarette Smoke Extract, (H) $NaIO_3$: Sodium Iodate, (I) VEGF-A: Recombinant human Vascular Endothelial Growth Factor-A, and (J) OPN: Recombinant Osteopontin (Scale bar = 200 μm).
